# Taxonomic Notes on *Nasutitermes* and *Bulbitermes* (Termitidae, Nasutitermitinae) from the Sunda region of Southeast Asia based on morphological and molecular characters

**DOI:** 10.3897/zookeys.148.2055

**Published:** 2011-11-21

**Authors:** Syaukani, Graham J. Thompson

**Affiliations:** 1 Department of Biology, Faculty of Mathematics and Natural Sciences, Syiah Kuala University, Darussalam 23111, Banda Aceh, Indonesia; 2 Department of Biology, Faculty of Science, University of Western Ontario, 1151 Richmond Road North, London N6A 5B7, Ontario, Canada

**Keywords:** Isoptera, Southeast Asia, morphological key, DNA barcodes, barcode gap

## Abstract

The Sunda region of Southeastern Asia is rich in termite fauna, but termites from this region have been poorly described. In this study, we described eight species from two diverse genera from this region, and from the family Termitidae. We describe *Bulbitermes* 4 spp. and *Nasutitermes* 4 spp. from new field collections. Where possible we examine original holotype specimens, and describe the essential morphological characters for soldier and worker castes. We devise two new bifurcating keys to guide the field identification of each species. In addition, we develop a nucleotide sequence profile for the COI gene. From this molecular character matrix, we use Neighbour-Joining analysis to test the monophyly of each morphospecies and genus. We find that the morphological and molecular characters are highly concordant, whereby all taxa appear to represent distinct molecular clades. For termites, there is therefore agreement between the morphological taxonomic characters used to sort species from a bifurcating key and the molecular taxonomic characters used to sort species on a bifurcating tree. This joint analysis suggests that DNA barcoding holds considerable promise for termite taxonomy, especially for diverse clades like *Bulbitermes* and *Nasutitermes* for which a global morphological key would be intractable.

## Introduction

As the largest subfamily among the higher termites (Family Termitidae), the Nasutitermitinae include more than 650 species from over 80 genera (Kambhampati and Eggleton 2000). This diversity within a single subfamily creates some taxonomic challenges for delineating between species and among genera, and there are presumably additional taxa not yet described. In this study we describe representative taxa from two of the largest genera within Nasutitermitinae, *Nasutitermes* (4 spp.) and *Bulbitermes* (4 spp.), as represented from newly collected material from the Sunda region of Southeast Asia.

Globally, the Nasutitermitinae have a broad dispersion. Genera from this subfamily are present in all biogeographical regions except the palearctic (Pearce and Waite 1994), and include at least 16 genera that are endemic to Southeast Asia (Tho 1992). Relative to other biogeographic regions, the taxonomy of Nasutitermitinae in Southeast Asia remains poorly understood (Chhotani 1987, 1997; Gathorne-Hardy 2001). This may stem in part from the historically poor taxon sampling from within this region (Eggleton 1999), but may also stem from a lack of diagnostic characters that clearly distinguish some of the genera.

This lack of morphological variation also hinders phylogeny. One outstanding question is the affinity between *Nasutitermes* and *Bulbitermes* (Collins 1989; Jones and Brendell 1998; Tho 1992; Gathorne-Hardy 2001). Are these genera phylogenetically distinct, or do they represent a synonymy that should be re-classified? *Bulbitermes* was separated from *Nasutitermes* by Emerson (in Snyder 1949). However, this distinction is based mostly on negative criteria – for example, *Bulbitermes* is a wood-feeding nasute genus that has constrictions behind the antennal sockets on the soldiers and that is not *Longipeditermes*, *Lacessitermes*, *Hospitalitermes* or *Ceylonitermes* (Gathorne-Hardy 2001). For this reason, *Nasutitermes* and *Bulbitermes* remain poorly distinguished based on morphology, and these genera may even be paraphyletic.

In this report we present the taxonomic notes for representative *Bulbitermes* and *Nasutitermes* species collected from Sumatra and Malay Peninsula (i.e., the Sunda region) within Southeast Asia. Specifically, we use morphological characters to describe eight species, and devise a key for their field identification. Further, we use molecular sequence information from the mitochondrial gene ‘barcode’ region to test the idea that *Bulbitermes* and *Nasutitermes* each form monophyletic and evolutionarily distinct genera.

## Materials and methods

### Collection procedure

All specimens were collected from the field using a mix of random and systematic transect searches. For transect sampling we adopted a standardized protocol (Jones and Eggleton 2000). We laid straight belt-transects (100 × 2 m) at random through the forest. Each transect was divided into 20 five-meter sections, and each section was searched by one person for one hour. For each section, 12 samples of surface soil with associated leaf litter and woody debris were scraped up and carefully examined for termites (approximate size of surface sample, 50 cm^2 ^× 5 cm deep). Finally, tree trunks and buttress roots were also examined for the presence of termites. Particular attention was paid to the deep accumulations of litter and organic-rich soil between buttresses. Also, any carton sheeting or runways suggesting the presence of live termites were examined, up to a height of two meters. For random sampling, we simply used our best judgment to search for termites within the above types of habitat, but without the use of transects. Wherever possible, all castes and both sexes were sampled. In total, termites included in this study were sampled from multiple regions in Sunda region from 1999–2010.

### Morphological analysis

Morphological character terminology used for describing soldiers and workers follows the convention of Roonwal and Chhotani (1989) and Sands (1998). For all species, we photographed the heads, bodies (in profile) and pronota of the soldier caste using a high-quality digital microscope (HFVH-8000, KEYENCE, Osaka). Further, from select samples we removed the antennae from the soldier caste and the mandibles of the worker caste. We then examined these diagnostic characters on glass slides mounted with Euparal 3C 239 (Waldeck GmbH & Co. KG, Muenster Germany). We photographed the mounts using a conventional digital camera (Coolpix 3340, Nikon, Tokyo) attached to a Nikon Eclipse E600 lens. From these images, we constructed a multi-focused montage using Helicon Focus 4.03 Pro software (Helicon Soft Ltd. Kharkov).

### Molecular barcode analysis

To aid with taxonomic analysis, we developed a cytochrome c oxidase I gene (COI) profile. The profile consists of nucleotide sequence from the ‘barcode’ region of the mitochondrial genome (Herbert et al. 2003). First, we removed single legs from representative specimens and used in-house protocols at the Canadian Center for DNA Barcoding (Guelph, Ontario) to extract DNA (Ivanova et al. 2006) and PCR-amplify (Ivanova and Grainer 2007) the barcode region using the LCO1490t1 / HCO2198t1 primer combination. Finally, we sequenced the resultant 658 base pair (bp) fragment in both directions using the M13R/M13F primer pair. For sequencing, we used an ABI 3730XL automated sequencer and associated software (Applied Biosystems). From forward and reverse sequences, we generated a single consensus sequence using CodonCode Aligner v. 3.0.2 (CodonCode Corporation). We have deposited all sequence trace files and detailed specimen records, including precise sampling localities and GenBank accession numbers, onto the Barcode of Life Data System (BOLD; Ratnasingham and Hebert 2007) under the Project name ‘Termites of Indonesia’ with Project code TINDS.

Using the BOLD on-line workbench, we aligned nucleotide sequences and calculated a pairwise Kimura-2-parameter (K2P) distance matrix. For each species in the matrix, we calculated the minimum, mean and maximum genetic distance. We also calculated the mean nearest neighbour distance (average distance to the most closely related species). From these data, we tested for the presence of a ‘barcode gap’ - a disjunction between levels of intraspecific and interspecific variability–by plotting maximum intraspecific distance against mean distance to nearest neighbour (NN). A gap is evident from this plot when the NN distance consistently exceeds the intraspecific distance. Finally, we used the K2P distance matrix to build a Neighbour-Joining (NJ) Taxon ID tree.

## Results

### Species descriptions

For all species described below, we provide comparative images of key morphological traits. For *Bulbitermes*, soldiers are profiled in Figures 1–4 and workers (mandibles) are profiled in Figures 9–16. For *Nasutitermes*, soldiers are profiled in Figures 5–8, and workers (mandibles) are profiled in Figures 17–24.

**Figure:**
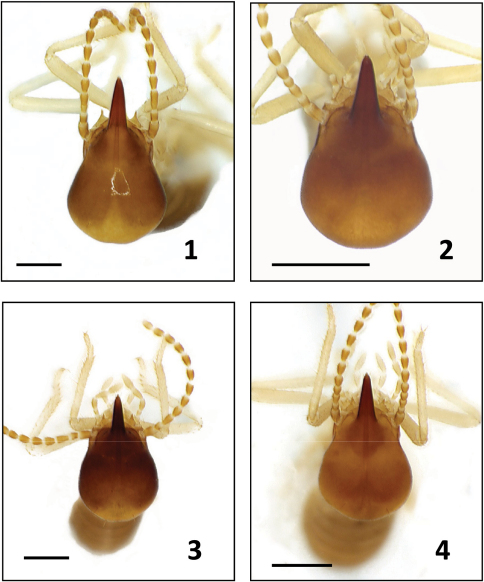
**Figures 1–4.** Soldiers of Bulbitermes from Sunda region in dorsal view. B. constrictus **1** B. singaporiensis **2** B. flavicans **3** B. neopusillus **4**

**Figure:**
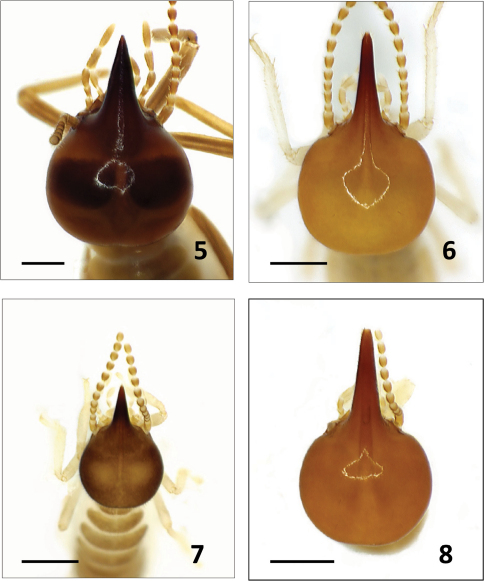
**Figures 5–8.** Soldiers of Nasutitermes from Sunda region in dorsal view. N. matangensis **5** N. longinasoides **6** N. neoparvus **7** N. longinasus (largest soldier) **8** Scale bar: 0.5 mm. Scale bar: 0.6 mm.

**Figure:**
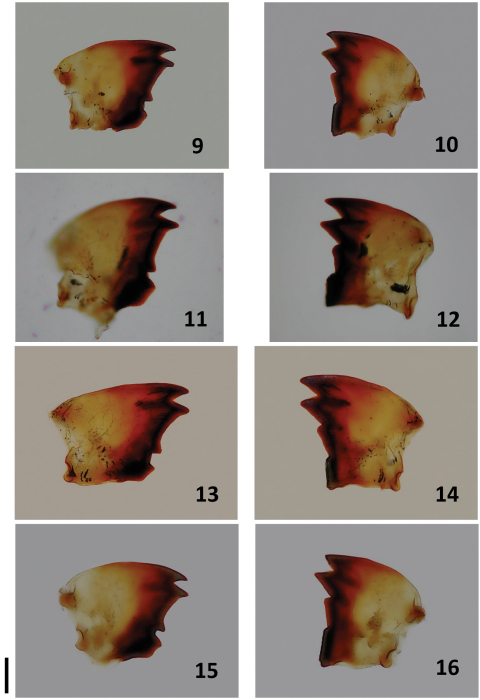
**Figures 9–16.** Workers of Bulbitermes from Sunda region. Left **9, 11, 13,** **15** and right **10, 12, 14, 16** mandibles. B. constrictus **9, 10** B. singaporiensis **11, 12** B. flavicans **13, 14** B. neopusillus **15, 16**. Scale bar: 0.1 mm.

**Figure:**
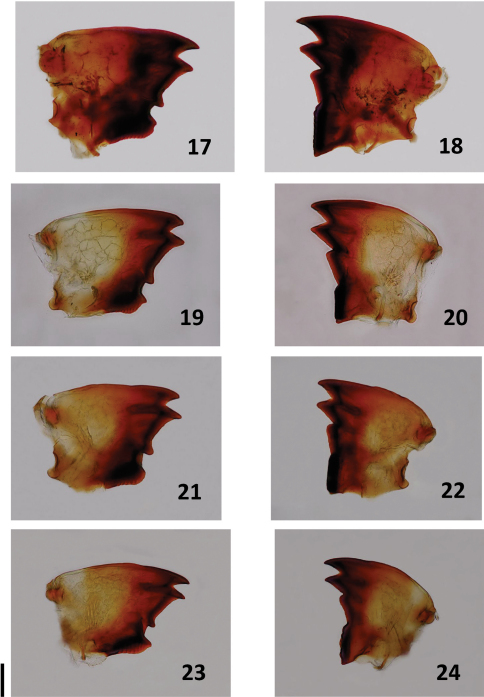
**Figures 17–24.** Workers of Nasutitermes from Sunda region. Left **17, 19**, **21, 23** and right **18, 20, 22, 24** mandibles. N. matangensis **17, 18** N. longinasoides **19, 20** N. neoparvus **21, 22** N. longinasus **23, 24**. Scale bar: 0.1 mm.

#### Bulbitermes flavicans

(Holmgren)

http://species-id.net/wiki/Bulbitermes_flavicans

Euternes (Euternes) flavicans Holmgren 1913, pp. 173–174.
Euternes (Euternes) flavicans : John 1925, p. 394.
Bulbitermes flavicans : Snyder 1949, p. 308.
Bulbitermes flavicans : 1958, p. 132 (key).
Bulbitermes flavicans : Thapa 1981, pp. 335-337.
Bulbitermes flavicans : Tho 1992, p.159.

##### Material examined.

 Syntype: soldier, West Sumatra, Harau, Pajacombo, 17.iii.1913, Oscar John Coll. B.M. 1926–242 BMNH103898. Other material: SYK1999&2001-L-0146, 1453–56, 1463, 1465, 1467. Soldiers and workers from undisturbed forests, 1,000–1,400 m altitude, Kemiri Mountain, Southeast Aceh; SYK1999&2001-L-1457–60, 1462. Soldiers and workers from undisturbed forests, 150–350 m altitude, Bukit Lawang, Langkat, North Sumatra; SYK2000-L-1466. Soldiers and workers from undisturbed forest, 500 m altitude, Ketambe, Southeast Aceh; SYK2006-AL-0001, 0777, 1613. Soldiers and workers from disturbed. SYK2010-KTB-011, 027, 051, 098. Soldiers and workers from undisturbed forest, 300–500 m altitude, Ketambe Research Station, Ecosystem Leuser, Sumatra.

##### Description.

Imago: Unknown.

Soldiers.Head: in dorsal view the anterior part darker than posterior part in coloration; rostrum dark brown with the apex paler; antenna much paler than anterior part of head capsule, uniformly coloured. Head with five or six scattered bristles, tip of nasus with four bristles, pronotum and abdominal tergites with microscopic hairs.Head capsule somewhat round, weakly constricted behind antennal sockets; posterior margin roundly convex; dorsal outline (including rostrum) in profile nearly straight with two shallow indentations near base of rostrum, and up-curved apically. Mandible with weak apical processes. Antenna with 12 articles; second clearly shorter than third and fourth; third clearly longer than fourth; fourth and fifth almost equal in length. Thorax: pronotum seen from above paler than anterior part of head capsule; its periphery paler than central area. Coxae pale brown; femora yellowish; tibiae whitish yellow. Anterior margin of pronotum nearly straight; posterior margin roundly convex. Abdomen: tergites pale brown.

Soldiers (n = 10; (size range) in mm): head length including nasus (HLN) (1.22–1.40); head length measured to base of mandible (HL) (0.97–1.00); nasus length (NL) (0.30–0.42); nasus index = NL/HL (0.30–0.42); maximum head width at posterior part (HWP) (0.75–0.84); maximum height of head excluding postmentum (HH) (0.57–0.65); pronotum length (PL) (0.15–0.17); pronotum width (PW) (0.37–0.45).

Workers. Antenna: whitish yellow to yellow; 13 articles; second shorter than third; third clearly longer than fourth; fourth wider than fifth. Left mandible: apical tooth and first marginal tooth almost equal in length; third marginal moderately protruding from cutting edge; fourth almost completely hidden behind molar prominence. Right mandible: posterior edge of second marginal tooth nearly straight; inner layer of molar plate undeveloped and notch at proximal end of molar plate obsolete.

##### Geographical distribution.

 Sumatra, Peninsular Malaysia and Borneo.

#### Bulbitermes neopusillus

Snyder & Emerson

http://species-id.net/wiki/Bulbitermes_neopusillus

Bulbitermes neopusillus Snyder & Emerson, 1949, p. 309.
Bulbitermes neopusillus : Ahmad 1958, p.133 (key).

##### Material examined. 

Soldiers,Sumatra, Siak, 9.ii.1913, Oscar John Coll. B.M. 1926-242 BMNH103902. SYK2006-KSNP-0032, 0034, 0047. Soldiers and workers from disturbed forest, 500 m altitude, Air Hangat, Kerinci, Jambi; SYK1999-L-1409. Soldiers and workers from undisturbed forest, 1,250 m altitude, Kemiri Mountain, Southeast Aceh; SYK1999-L-1418, 1632, 1646, 1651, 1682. Soldiers and workers from undisturbed forest, 200-400 m altitude, Bengkung, Southeast Aceh; SYK1999&2000-L-1412, 1420, 1426, 1434, 1437, 1441, 1444, 1604, 1608, 1636, 1639, 1645, 1654, 1657, 1659, 1668, 1669. Soldiers and workers from undisturbed forest, 150-350 m altitude, Bukit Lawang, Langkat, North Sumatra; SYK1999-L-1405, 1442, 1448, 1612. Soldiers and workers from disturbed forest, 50 m altitude, Sekundur, Langkat, North Sumatra; SYK2000-L-1398, 1849. Soldiers and workers from disturbed forest, 80 m altitude, Soraya, Singkil, Aceh; SYK2000&2001-L-1394, 1395, 1415. Soldiers and workers from undisturbed forest, 300–500 m altitude, Ketambe, Southeast Aceh; SYK2006-AL-0778, 0779, 0780. Soldiers and workers from disturbed forest, 30 m altitude, Maestong, Sungai Batang Hari, Jambi; SYK2007-SPR-0038. Soldiers and workers from disturbed forest, <100 m altitude, Tua Pejat, Sipora Island, Mentawai, West Sumatra. SYK2010-KTB-003, 004, 005, 018, 035, 044, 045, 047, 048, 054, 055, 056, 057, 059, 060, 065, 066, 067, 068, 071, 075. Soldiers and workers from undisturbed forest, 300–500 m altitude, Ketambe Research Station, Ecosystem Leuser, Sumatra.

##### Description.

 Imago: Unknown.

Soldiers. Head: in dorsal view anterior and posterior parts almost similar in coloration; rostrum paler than anterior part of head capsule. Antenna paler than anterior part of head capsule in coloration, with the first segment darkest.Head with two scattered bristles, tip of nasus with four bristles, pronotum and abdominal tergites with microscopic hairs. In dorsal view head capsule somewhat pear-shaped, strongly constricted behind antennal sockets; its posterior margin weakly indented in middle; dorsal outline (including rostrum) in profile nearly straight. Mandible with short apical processes. Antenna with 13 articles; second shorter than third; third twice as long as fourth; fourth shorter than fifth. Thorax: pronotum seen from above similar to the posterior part of head capsule in coloration; its periphery darker than central area. Coxae and femora pale brown; tibiae pale yellow. Anterior and posterior margins of pronotum nearly straight. Abdomen: tergites pale brown to brown.

Soldiers (n = 10; (size range) in mm): head length including nasus (HLN) (1.45–1.60); head length measured to base of mandible (HL) (0.90–0.93); nasus length (NL) (0.55–0.62); nasus index = NL/HL (0.62–0.67); maximum head width at posterior part (HWP) (0.85–0.87); maximum height of head excluding postmentum (HH) (0.60–0.63); pronotum length (PL) (0.15–0.18); pronotum width (PW) (0.50–0.55).

Workers. Antenna: whitish yellow; 14 articles; second longer than third and fourth; third clearly longer than fourth; fourth the shortest; fifth shorter than sixth. Left mandible: apical tooth shorter than first marginal tooth; third marginal tooth moderately protruding from cutting edge; fourth marginal tooth hidden behind molar prominence. Right mandible: posterior edge of second marginal tooth nearly straight; inner layer of molar undeveloped and notch at proximal end of molar plate weakly developed.

##### Geographical distribution.

 Sumatra, Mentawai Islands, Peninsular Malaysia, Java and Borneo.

#### Bulbitermes constrictus

(Haviland)

http://species-id.net/wiki/Bulbitermes_constrictus

Termes constrictus Haviland, 1898, pp. 420–421.
Eutermes (Eutermes) constrictus : Holmgren 1913, p. 172.
Bulbitermes constrictus : Snyder 1949, p. 308.
Bulbitermes constrictus : Ahmad 1958, pp. 129, 133–134 (key).
Bulbitermes constrictus : Thapa 1981, pp. 332–335.

##### Material examined.

 Syntype:Soldiers, Sarawak, Coll.& Det. G.D. Haviland, No. 292, (Ex. Dundee) (Bulbitermes) MNH103897. Other material: SYK2006-KSNP-0006, 0083. Soldiers and workers from undisturbed forest, 300 m altitude, Sungai Manau, Merangin, Jambi; SYK2006-KSNP-0081, 0090. Soldiers and workers from disturbed forest, 500 m altitude, Air Hangat, Kerinci, Jambi; SYK2000-L-1507, 1522. Soldiers and workers from undisturbed forest, 250–350 m altitude, Bukit Lawang, Langkat, North Sumatra; SYK2000&2001-L-1496, 1497, 1498. Soldiers and workers from disturbed forest, 50 m altitude, Sekundur, Langkat, North Sumatra. SYK2010-KTB-013, 014, 015, 137, 038, 046, 049, 050, 100. Soldiers and workers from undisturbed forest, 300–500 m altitude, Ketambe Research Station, Ecosystem Leuser, Sumatra.

##### Description.

 Imago: Unknown.

Soldiers. Head: in dorsal view anterior and posterior parts almost similar in coloration; except around posterior margin, much paler in coloration; rostrum darker than anterior part of head capsule; antenna paler than anterior part of head capsule in coloration. Head with two bristles, tip of nasus with four bristles, pronotum with brittles, abdominal tergites with hairs and bristles.

In dorsal view head capsule somewhat pear-shaped, strongly constricted behind antennal sockets; its posterior margin weakly indented; dorsal outline (including rostrum) in profile nearly straight. Mandible with moderately developed apical processes. Antenna with 14 articles; second longer than third; third the shortest; fourth and fifth almost equal in length. Thorax: pronotum seen from above paler than anterior part of head capsule; its periphery darker than central area. Coxae and femora yellow; tibiae pale yellow. Anterior margin of pronotum weakly indented in the middle, while posterior margin roundly convex. Abdomen: tergites yellowish to pale brown.

Soldiers (n = 10; (size range) in mm): head length including nasus (HLN) (1.55–1.65); head length measured to base of mandible (HL) (1.05–1.13); nasus length (NL) (0.50–0.60); nasus index = NL/HL (0.47–0.53); maximum head width at posterior part (HWP) (0.95–0.10); maximum height of head excluding postmentum (HH) (0.60–0.65); pronotum length (PL) (0.22-0.25); pronotum width (PW) (0.52–0.55).

Workers. Antenna: whitish yellow to yellow; 15 articles; second clearly longer than third; third the shortest; fourth and fifth almost equal in length. Left mandible: apical and first marginal teeth almost equal in length; third marginal moderately protruding from cutting edge; fourth almost completely hidden behind molar prominence. Right mandible: posterior edge of second marginal tooth nearly straight; inner layer of molar not developed; notch at proximal end of molar plate weakly developed.

##### Geographical distribution.

 Sumatra and Borneo.

#### Bulbitermes singaporiensis

(Haviland)

http://species-id.net/wiki/Bulbitermes_singaporiensis

Termes singaporiensis Haviland, 1898, pp. 429.
Eutermes (Eutermes) singaporiensis : Holmgren 1913, p. 179.
Bulbitermes singaporiensis : Snyder 1949, p. 309.
Bulbitermes singaporiensis : Tho 1992, pp. 158–159.

##### Material examined.

 Syntype: soldiers,*Termes singaporiensis*, Singapore, 1893, Coll. & Det. G. D. Haviland, No. 16 (Ex. Dundee) (Bulbitermes) BMNH103923. Other material: SYK(1998–2001)-L-0205, 0212, 0219, 1802, 1812, 1817, 1821–23, 1825–29, 1831, 1832, 1834, 1835, 1839, 1840, 1843, 1846–48, 1851, 1853–55, 1859, 1860, 1865, 1867, 1868, 1870, 1877, 1883, 1884, 1850, 1877, 1879, 1880, 1975, 1978. Soldiers and workers from undisturbed forest, 150–350 m altitude, Bukit Lawang, Langkat, North Sumatra.; SYK2006-AL-0004, 0801, 0802, 1886. Soldiers and workers from disturbed forest, 30 m altitude, Maestong, Sungai Batang Hari, Jambi; SYK2006-L1852. Soldiers and workers from undisturbed forest, 50 m altitude, Sekundur, Langkat, North Sumatra; SYK1999-L-1882. Soldiers and workers from undisturbed forest, 400 m altitude, Bengkung, Southeast Aceh. SYK&FAZLY2009-ER-020, 022, 024, 039, 043, 044, 047, 051, 057, 058, 062, 063, 087. Soldiers and workers from undisturbed forest, >100 m altitude, Endau Rompin National Park, Johor, Peninsular Malaysia.

##### Description.

Imago: Unknown.

Soldiers. Head: in dorsal view anterior part of head capsule darker than the posterior part in coloration; rostrum slightly darker than anterior part of head capsule; antenna paler than anterior part of head capsule. Head with two bristles, tip of nasus with four bristles, pronotum with microscopic hairs, abdominal tergites with hairs and bristles.In dorsal view head capsule somewhat pear-shaped and weakly constricted behind antennal sockets; its posterior margin nearly straight in the middle; dorsal outline (including rostrum) in profile nearly straight; mandible with moderately developed apical processes. Antenna with 12 articles; second and third almost equal in length, the former wider than the latter in width; third the shortest; fourth and fifth almost equal in length. Thorax: pronotum seen from above paler than anterior part of head capsule; its anterior part slightly darker than posterior part. Coxae pale brown; femora yellowish; tibiae pale yellow. Anterior margin of pronotum moderately indented in the middle, while posterior margin nearly straight in the middle. Abdomen: tergites dark brown to very dark sepia brown.

Soldiers (n = 10; (size range) in mm): head length including nasus (HLN) (1.24–1.35); head length measured to base of mandible (HL) (0.98–1.12); nasus length (NL) (0.32–0.37); nasus index = NL/HL (0.31–0.35); maximum head width at posterior part (HWP) (0.80–0.84); maximum height of head excluding postmentum (HH) (0.57–0.61); pronotum length (PL) (0.16–0.18); pronotum width (PW) (0.37–0.44).

Workers. Antenna: whitish yellow to yellow with first article darker than the subsequent; 14 articles; second much longer than third and fourth; third longer than fourth; fourth the shortest. Left mandible: apical tooth shorter than first marginal tooth; third marginal moderately protruding from cutting edge; fourth almost completely hidden behind molar prominence. Right mandible: posterior edge of second marginal tooth weakly concave; inner layer of molar plate and notch at proximal end of molar plate obtuse.

##### Biogeographical distribution.

 Sumatra, Peninsular Malaysia, Java and Borneo.

#### Nasutitermes matangensis

(Haviland)

http://species-id.net/wiki/Nasutitermes_matangensis

Termes matangensis Haviland, 1898, pp. 427–428.
Eutermes (Eutermes) matangensiformis : Holmgren 1913, p. 185.
Eutermes (Eutermes) matangensis : Holmgren 1913-14, pp. 26–265.
Eutermes (Eutermes) matangensis : John 1925, p. 398.
Nasutitermes matangensis : Snyder 1949, p. 287.
Nasutitermes matangensis : Ahmad 1958, p. 147(key).
Nasutitermes matangensis : Thapa 1981, pp. 312–315.
Nasutitermes matangensis : Tho 1992, p. 159.

##### Material examined.

Syntype:Soldiers, *Termes matangensis*, Sarawak, Coll. G. D. Haviland. BMNM No. 358. (Ex. Dundee) (*Nasutitermes*). Other material: SYK2006-KSNP-0017, 0023, 0025, 0041. Soldiers and workers from undisturbed forest, 300 m altitude, Sungai Manau, Merangin, Jambi; SYK1999&2001-L-1039, 1040, 1042, 1043, 1045, 3080. Soldiers and workers from undisturbed forest, 1,000–1,400 m altitude, Kemiri Mountain, Southeast Aceh; SYK2007-UGDT-0010, 0063–70. Soldiers and workers from disturbed forest, 900 m altitude, Ulu Gadut, Padang, West Sumatra; SYK2007-SRSR-0005, 0040, 0063, 0066, 0632. Soldiers and workers from disturbed forest, <100 m altitude, Siberut Tengah, Mentawai, West Sumatra; SYK2007-SBRT-0016, 0034, 0039, 0046, 0047, 0060, 0070, 0080. Soldiers and workers from undisturbed forest, <100 m altitude, Simabuggei, Siberut Island, Mentawai, West Sumatra; SYK2001-S-0128, 0030. Soldiers and workers from disturbed forest, 200 m altitude, Aceh Besar; SYK2006-SB-0001–04, 0044, 0045, 0050, 0051. Soldiers and workers from disturbed forest, <100 m altitude, Sabang, Weh Island, Aceh; SYK1999-L-1062. Soldiers and workers from undisturbed forest, 300 m altitude, Ketambe, Southeast Aceh; SYK2006-AL-0901. Soldiers and workers from disturbed forest, 30 m altitude, Maestong, Sungai Batang Hari, Jambi; SYK2002-NIAS-0111. Soldiers and workers from disturbed forest, <100 m altitude, Gunung Sitoli, Nias Island, North Sumatra; SYK2002-PRP-0100. Soldiers and workers from disturbed forest, 900 m altitude, Toba Lake, Parapat, North Sumatra; SYK2007-LP-0093. Soldiers and workers from disturbed forest, 1100 m altitude, Sumber Jaya, Kota Bumi, Lampung; SYK2007-LBAN-0004, 0012. Soldiers and workers from disturbed forest, 600 m altitude, Lembah Anai, Tanah Datar, West Sumatra; SYK2005&2006-RKT-0002, 0004, 0006, 0007, 0009–0021, 0033–40, 0042–48, 0050, 0055, 0056, 0058, 0059, 0061–0063, 0065, 0066, 0072. Soldiers and workers, <10–300 m altitude, Rakata island, the Krakataus, Lampung; SYK2005&2006-ANK-0040, 0041, 0043, 0139, 0144–46, 0148, 0149, 0151, 0154, 0155, 0277–79, 0301, 0302. Soldiers and workers, <100 m altitude, Anak Krakatau, the Krakataus, Lampung; SYK2005&2006-PJG-0073–0083, 0092–0095, 0137, 0138, 0141, 0142, 0147, 0150–0153, 0156, 0157. Soldiers and workers, <100 m altitude, Panjang island, the Krakataus, Lampung; SYK2005&2006-SRTG-0006, 0120, 0126–30, 0041–44, 0173–90, 0192–95, 0197–200, 0216, 0227–30, 0238. Soldiers and workers, <100 m altitude, Sertung island, the Krakataus, Lampung; SYK2005-SBK-0235, 0237–42, 0246, 0263. Soldiers and workers, <100 m altitude, Sebuku island, the Krakataus, Lampung; SYK2005-SBS- 0064, 0067, 0069–71, 0073–75, 0077–88, 0090. Soldiers and workers, <100 m altitude, Sebesi island, the Krakataus, Lampung; SYK2007-LGD-0031, 0050–52, 0058, 0059. Soldiers and workers, <100 m altitude, Legundi island, the Krakataus, Lampung; SYK2007-LP-0002, 0006. Soldiers and workers, from disturbed forest, <100 m altitude, Pantai Mutun, Lampung. SYK2010-KTB-002, 006, 019, 041, 095. Soldiers and workers from undisturbed forest, 300–500 m altitude, Ketambe Research Station, Ecosystem Leuser, Sumatra; SYK2010-PK-005, 006. Soldiers and workers from disturbed forest, 150 m altitude, Peukan Biluy, Darul Kamal, Aceh Besar, Sumatra; SYK&FAZLY2009-ER-038, 050, 073. Soldiers and workers from undisturbed forest, >100 m altitude, Endau Rompin National Park, Johor, Peninsular Malaysia.

##### Description.

Imago: Unknown.

Soldiers. Head: in dorsal view head capsule excluding rostrum brown to dark sepia brown; rostrum darker than head capsule; entire rostrum almost uniformly coloured; antenna paler than head capsule in coloration. Head with a few number of scattered bristles, tip of nasus with four bristles, pronotum hairs, abdominal tergites with hairs and brittles. In dorsal view head capsule round; its posterior margin nearly straight in the middle; dorsal outline (including rostrum) in profile nearly straight, down-curved apically. Antenna with 13 articles; second slightly longer than fourth; third the longest; fourth shorter than fifth. Thorax: pronotum seen from above paler than head capsule in coloration; its anterior part darker than posterior part. Coxae and femora sepia brown; tibiae yellowish. Anterior margin of pronotum moderately indented in the middle; posterior margin nearly straight in the middle. Abdomen: tergites yellowish to pale brown.

Soldiers (n = 10; (size range) in mm): head length including nasus (HLN) (2.05–2.18); head length measured to base of mandible (HL) (1.20–1.25); nasus length (NL) (0.82–0.95); nasus index = NL/HL (0.68–0.76); maximum head width (HW) (1.37–1.50); maximum height of head excluding postmentum (HH) (0.90–0.95); pronotum length (PL) (0.25–0.30); pronotum width (PW) (0.60–0.70).

Workers. Antenna: pale yellow to yellow; 14 articles; second clearly longer than third and fourth; third longer than fourth. Left mandible: apical tooth longer than first marginal tooth; third marginal moderately protruding from cutting edge; fourth almost completely hidden behind molar prominence. Right mandible: posterior edge of second marginal tooth nearly straight; inner layer of molar plate undeveloped; notch at proximal end of molar plate absent.

##### Geographical distribution.

 Sumatra, Mentawai islands, Malay Peninsula, Java and Borneo.

#### Nasutitermes neoparvus

Thapa

http://species-id.net/wiki/Nasutitermes_neoparvus

Nasutitermes neoparvus Thapa 1981, pp. 329–332.

##### Material examined.

 SYK2006-KSNP-0007, 0053. Soldiers and workers from undisturbed forest, 300 m altitude, Sungai Manau, Merangin, Jambi; SYK2006-KSNP-0054, 0076, 0085. Soldiers and workers from disturbed forest, 500m altitude, Air Hangat, Kerinci, Jambi; SYK2007-SRSR-0006, 0011. Soldiers and workers from disturbed forest, <100 m altitude, Surisura, Siberut Tengah, Mentawai, West Sumatra; SYK2006-SBRT-0006, 0028, 0077, 0084, 0103. Soldiers and workers from undisturbed forest, <100 m altitude, Simabuggei, Siberut island, Mentawai, West Sumatra; SYK1999&2000-L-0916, 0923, 0931, 0932, 0935, 0937, 0940, 0943. Soldiers and workers from undisturbed forest, 150–350 m altitude, Bukit Lawang, Langkat, North Sumatra; SYK2000&2001-L- 0214, 0913, 0918, 0922, 0926, 0928, 0933, 0934, 0941, 0942. Soldiers and workers from disturbed forest, 50 m altitude, Sekundur, Langkat, North Sumatra; SYK1999-L-0208, 0235, 0241, 0930, 0917, 0921, 0924, 0936, 0939. Soldiers and workers from undisturbed forest, 200–400 m altitude, Bengkung, Southeast Aceh; SYK1999-L-0211, 0225, 0911, 0914, 0915, 0919, 0920, 0925, 0927, 0929, 0938, 1171. Soldiers and workers from disturbed forest, 80 m altitude, Soraya, Singkil, Aceh; SYK1999-L- 0912, 0215, 0237, 0244, 0245. Soldiers and workers from undisturbed forest, 300–500 m altitude, Ketambe, Southeast Aceh; SYK2007-LP-0081. Soldiers and workers from disturbed forest, <100 m altitude, Pantai Mutun, Lampung. SYK2010-KTB-029, 053, 058, 061. Soldiers and workers from undisturbed forest, 300–500 m altitude, Ketambe Research Station, Ecosystem Leuser, Sumatra; SYK&FAZLY2009-ER-034. Soldiers and workers from undisturbed forest, >100 m altitude, Endau Rompin National Park, Johor, Peninsular Malaysia.

##### Description.

Imago: Unknown.

Soldiers. Head: in dorsal view head capsule excluding rostrum dark brown to sepia brown; rostrum darker than head capsule; antenna much paler than head capsule. Head with combination long and short bristles, tip of nasus with short hairs and four long bristles, pronotum with short and long bristles, abdominal tergites densely with short hairs and long bristles.In dorsal view head capsule somewhat round; its posterior margin roundly convex in the middle; dorsal outline of head capsule (excluding rostrum) in profile nearly straight. Antenna with 11 articles; second shorter than fourth; third longer than fourth; fourth and fifth almost equal in length. Thorax: pronotum seen from above paler than head capsule in coloration; its periphery darker than central area. Coxae and femora yellow; tibiae whitish yellow. Anterior margin of pronotum nearly straight in the middle; posterior margin strongly indented in the middle. Abdomen: tergites dark yellow to brown.

Soldiers (n = 10; (size range) in mm): head length including nasus (HLN) (1.25–1.37); head length measured to base of mandible (HL) (0.62–0.77); nasus length (NL) (0.57–0.62); nasus index = NL/HL (0.79–0.80); maximum head width (HW) (0.77–0.80); maximum height of head excluding postmentum (HH) (0.50–0.60); pronotum length (PL) (0.12–0.15); pronotum width (PW) (0.37–0.42).

Workers. Antenna: pale yellow to yellow; 12 articles; second clearly longer than fourth; third longer than fourth; fourth shorter than fifth. Left mandible: apical tooth clearly shorter than first marginal tooth; third marginal moderately protruding from cutting edge; fourth visible in the gap between third marginal and molar prominence. Right mandible: posterior edge of second marginal tooth nearly straight; inner layer of molar plate undeveloped, and notch at proximal end of molar plate obtuse.

##### Geographical distribution.

 Sumatra and Borneo.

#### Nasutitermes longinasus

(Holmgren)

http://species-id.net/wiki/Nasutitermes_longinasus

Eutermes (Eutermes) longinasus Holmgren 1913, p. 171.
Eutermes longinasus : John 1925, p. 392.
Nasutitermes longinasus : Snyder 1949, p. 284.
Nasutitermes longinasus : Ahmad 1958, p. 54 (key).
Nasutitermes longinasus : Thapa 1981, pp. 323-326.
Nasutitermes longinasus : Tho 1992, p. 150.

##### Material examined.

 SYK2006-KSNP-0040, 0049, 0052. Soldiers and workers from undisturbed forest, 300 m altitude, Sungai Manau, Merangin, Jambi; SYK(1998–2001)-L-0012, 0025, 0027, 0234, 0036, 0167, 0218, 0231, 0851, 0856, 1034, 1070, 1071, 1080, 1085, 1095. Soldiers and workers from disturbed forest, 80 m altitude, Soraya, Singkil, Aceh; SYK1999-L-0010, 0020, 0029, 0030, 0035, 0039, 0044, 0047–49, 0055, 0056, 0061, 0065, 0066, 0071, 0110, 0222, 0226, 0235, 0240, 0246, 0249, 0277, 0825, 0826, 0848–50, 0853–55, 0857, 0859–61, 1044, 1048, 1068, 1069, 1073, 1074, 1076, 1078, 1079, 1081–83, 1086, 1089–92, 1096, 1097. Soldiers and workers from undisturbed forest, 200–400 m altitude, Bengkung, Southeast Aceh; SYK1999&2000-L-0069, 0210, 0233, 0858, 1072, 1075, 1077, 1087. Soldiers and workers from undisturbed forest, 300–500 m altitude, Ketambe, Southeast Aceh; SYK(1999–2001)-L-0018, 0406, 1087, 1088, 1093. Soldiers and workers from undisturbed forest, 150–350 m altitude, Bukit Lawang, Langkat, North Sumatra. SYK2010-KTB-026, 028, 031, 032, 033, 034, 076, 077, 079, 082, 083, 084, 085, 086, 088, 091, 092, 093, 094, 096, 097. Soldiers and workers from undisturbed forest, 300–500 m altitude, Ketambe Research Station, Ecosystem Leuser, Sumatra.

##### Description.

Imago: Unknown.

Major soldiers. Head: in dorsal view head capsule excluding rostrum pale brown to brown; rostrum darker than head capsule; antenna much paler than head capsule in coloration. Head with hairs and two long bristles, tip of nasus with hairs and four brittles, pronotum with short and long bristles, pronotum and abdominal tergites with hairs and brittles. In dorsal view head capsule round; its posterior margin nearly straight in the middle; dorsal outline of head capsule (including rostrum) in profile nearly straight. Antenna with 13 articles; second longer than fourth; third clearly longer than fourth; fourth the shortest. Thorax: pronotum seen from above paler than head capsule in coloration; its periphery darker than central area. Coxae and femora yellow; tibiae whitish yellow. Anterior margin of pronotum moderately indented in the middle, while posterior margin roundly convex in the middle. Abdomen: tergites yellow to brown.

Major soldiers (n = 10; (size range) in mm): head length including nasus (HLN) (2.00–2.20); head length measured to base of mandible (HL) (1.07–1.15); nasus length (NL) (0.95–1.12); nasus index = NL/HL (0.88–0.97); maximum head width (HW) (1.15–1.30); maximum height of head excluding postmentum (HH) (0.80–0.85); pronotum length (PL) (0.25–0.30); pronotum width (PW) (0.52–0.58).

Minor soldiers: in dorsal view head capsule excluding rostrum deep reddish brown; rostrum same color as head capsule; antenna paler than head capsule in coloration. Head with hairs and two long bristles, tip of nasus with hairs and four bristles, pronotum with short and long bristles, pronotum and abdominal tergites with hairs and bristles. In dorsal view head capsule round; its posterior margin weakly constricted in the middle; dorsal outline of head capsule (including rostrum) in profile nearly straight. Antenna with 13 articles; second longer than fourth; third clearly longer than fourth; fourth the shortest. Thorax: pronotum seen from above slightly paler than head capsule in coloration; its periphery darker than central area. Coxae and femora brownish yellow; tibiae yellow. Anterior margin of pronotum weakly to moderately indented in the middle, while posterior margin roundly convex in the middle. Abdomen: tergites brown.

Minor soldiers (n = 10; (size range) in mm): head length including nasus (HLN) (172–1.82); head length measured to base of mandible (HL) (0.82–0.86); nasus length (NL) (0.71–0.77); nasus index = NL/HL (0.86–0.89); maximum head width (HW) (0.86–0.94); maximum height of head excluding postmentum (HH) (0.59–0.67); pronotum length (PL) (0.20- 0.23); pronotum width (PW) (0.49–0.51).

Workers. Antenna: pale yellow to yellow, with the first article darker than the subsequent; 14 articles; second and third clearly longer than fourth; fourth the shortest. Left mandible: apical tooth longer than first marginal tooth; third marginal weakly protruding from cutting edge; fourth hidden behind molar prominence. Right mandible: posterior edge of second marginal tooth nearly straight; inner layer of molar plate weakly developed; notch at proximal end of molar plate obtuse.

##### Geographical distribution.

 Sumatra, Peninsular Malaysia and Borneo.

#### Nasutitermes longinasoides

Thapa

http://species-id.net/wiki/Nasutitermes_longinasoides

Nasutitermes longinasoides Thapa 1981, pp. 301–303 .

##### Material examined.

 SYK2006-KSNP-0028, 0031, 0037, 0039, 0050, 0058. Soldiers and workers from undisturbed forest, 300 m altitude, Sungai Manau, Merangin, Jambi; SYK2006-AL-0016, 0021, 0106, 0950-0952. Soldiers and workers from disturbed forest, 30 m altitude, Maestong, Sungai batang hari, Jambi; SYK1999&2000-L-1063–1066. Soldiers and workers from undisturbed forest, 300–500 m altitude, Ketambe, Southeast Aceh; SYK2002-PRP-0101, 0102. Soldiers and workers from disturbed forest, 900 m altitude, Parapat, North Sumatra. SYK2010-KTB-012, 022, 030, 042, 043, 063, 069, 090. Soldiers and workers from undisturbed forest, 300–500 m altitude, Ketambe Research Station, Ecosystem Leuser, Sumatra.

##### Description.

Imago: Unknown.

Soldiers. Head: in dorsal view head capsule excluding rostrum pale yellow to yellow; rostrum darker than head capsule; antenna slightly paler than head capsule in coloration. Head with three or four scattered bristles, tip of nasus a hairs and four bristles, pronotum and abdominal tergites with hairs and brittles. In dorsal view head capsule round; its posterior margin roundly convex in the middle; dorsal outline of head capsule (including rostrum) in profile nearly straight. Antenna with 13 articles; second and fourth almost equal in length; third clearly longer than fourth; fourth shorter than fifth. Thorax: pronotum seen from above paler than head capsule in coloration; its periphery darker than central area. Coxae and femora pale yellow; tibiae whitish. Anterior and posterior margins of pronotum nearly straight in the middle. Abdomen: tergites yellow to pale brown.

Soldiers (n = 10; (size range) in mm): head length including nasus (HLN) (2.10–2.20); head length measured to base of mandible (HL) (1.07–1.25); nasus length (NL) (0.92–1.07); nasus index = NL/HL (0.85–0.86); maximum head width (HW) (1.23–1.32); maximum height of head excluding postmentum (HH) (0.80–0.83); pronotum length (PL) (0.20–0.23); pronotum width (PW) (0.56–0.60).

Workers. Antenna: pale yellow to yellow; 14 articles; second much longer than fourth; third longer than fourth; fourth the shortest. Left mandible: apical tooth shorter than first marginal tooth; third marginal moderately protruding from cutting edge; fourth completely hidden behind molar prominence. Right mandible: posterior edge of second marginal tooth nearly straight; inner layer of molar plate undeveloped, and notch at proximal end of molar plate obtuse.

##### Geographical distribution.

 Sumatra and Borneo.

## Taxonomic key

### Key 1. Key to the species of Bulbitermes based on the largest soldiers and largest workers

**Table:** 

1	Soldier: antenna with 12 articles	2
–	Soldier: antenna with 13 to 14 articles	3
2(1)	Worker: antenna with 13 articles, second shorter than third, fourth and fifth almost equal in length. Left mandible of apical tooth shorter than first marginal tooth	*Bulbitermes flavicans*
–	Worker: antenna with 14 articles, second article much longer than third, fourth article the shortest. Apical and first marginal teeth of left mandible almost equal in length	*Bulbitermes singaporiensis*
3(1)	Soldier: antenna with 13 articles, fourth article the shortest. In dorsal view posterior margin weakly indented the middle. Worker: antenna with 14 articles, fourth article the shortest	*Bulbitermes neopusillus*
–	Soldier: antenna with 14 articles, third article the shortest, In dorsal view posterior margin nearly straight. Worker: antenna with 14 articles, third the shortest	*Bulbitermes constrictus*

### Key 2. Key to the species of Nasutitermes based on the largest soldiers and largest workers

**Table:** 

1	Soldier: antenna with 11 articles. [Worker: antenna with 12 articles.]	*Nasutitermes neoparvus*
–	Soldier: antenna with 13 articles	2
2(1)	Soldier: dimorphic. [The largest and smallest individuals differ markedly in size.]	*Nasutitermes longinasus*
–	Soldier: monomorphic	3
3(2)	Soldier: in dorsal view posterior margin of head capsule roundly convex in the middle, dorsal outline of head capsule (including rostrum) in profile nearly straight, maximum width of head capsule 1.23-1.32 mm. Worker: apical tooth of left mandible shorter than first marginal tooth	*Nasutitermes longinasoides*
–	Soldier: in dorsal view posterior margin of head capsule nearly straight in the middle, dorsal outline (including rostrum) in profile nearly straight, down-curved apically, maximum width of head capsule 1.37-1.50 mm. Worker: apical tooth of left mandible longer than first marginal tooth	*Nasutitermes matangensis*

### Molecular barcode analysis

The study profile includes 48 COI sequences, representing eight putative species, as identified from the field using morphological characters. Of these taxa, there are four morphologically assigned to *Nasutitermes* and an additional four assigned to *Bulbitermes*. The number of specimens sequenced from each morphospecies ranged from 1 (a single individual) to 21, depending on specimen availability and sequencing success.

Using the built-in analysis workbench of BOLD, we built a Taxon ID tree using the NJ algorithm applied to a pair wise K2P distance matrix (Figure 25). This tree shows that *Nasutitermes* and *Bulbitermes* form monophyletic groups within the NJ analysis. Moreover, each morphospecies within each of these genera form monophyletic groups. No genera or morphospecies were paraphyletic with respect to each other. There is therefore a high degree of concordance between the morphological taxonomic characters used to sort species from a bifurcating key (Key 1, Key 2) and the molecular taxonomic characters used to sort species on a bifurcating tree (Figure 25).

**Figure:**
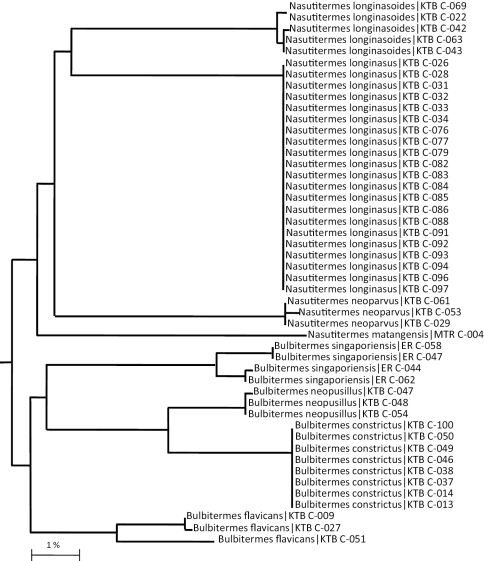
**Figure 25.** Neighbour-Joining analysis of K2P-corrected distances based on the analysis of 650 bp from the COI-5P marker locus in Nasutitermes spp. and Bulbitermes spp. collected from Sunda region.

Of the four *Bulbitermes* taxa examined, *Bulbitermes constrictus* forms a sister group with *Bulbitermes neopusillus*, which together form a sister group with *Bulbitermes singaporiensis*. This complex is in turn a sister group to *Bulbitermes flavicans*. Of the *Bulbitermes* taxa represented here, only *Bulbitermes constrictus* is invariant in the barcode region across all eight specimens analyzed.

Of the four *Nasutitermes* taxa examined, *Nasutitermes longinasus* forms a sister group with *Nasutitermes longinasoides*, which together form a sister group with *Nasutitermes neoparvus*. This three-species complex is in turn sister group to *Nasutitermes matagensis*. Of the *Nasutitermes* taxa examined, only *Nasutitermes longinasus* is invariant in the barcode region across all 21 specimens examined.

Levels of barcode variation within and between species showed a distinct barcode gap, whereby the mean interspecific divergence was significantly larger than the mean intraspecific divergence (0.09% vs. 11.86%; *t* = 93.98, d.f., = 586, *P* < 0.0001; Table 1). Within genera, the intraspecific distance ranged from as low as 0% (fixed sequences) in *Bulbitermes constrictus* and *Nasutitermes longinasus*, to a high of 5.11% in *Bulbitermes flavicans*.

**Table 1.** Genetic distance summary for barcode sequences generated from Nasutitermes and Bulbitermes from Sunda region.

Comparison	No. Comparisons		Genetic distance (%)	
		Minimum	Mean (S.E.)	Maximum
Within species	263	00	0.09 (0.03)	5.11
Within genera	325	5.42	11.86 (0.11)	16.01

As expected the NN distance between species was always greater than the maximum intraspecific distance (Fig. 26). For no species was there an overlap in the range of maximum intraspecific distance and the distance to nearest neighbour.

**Figure:**
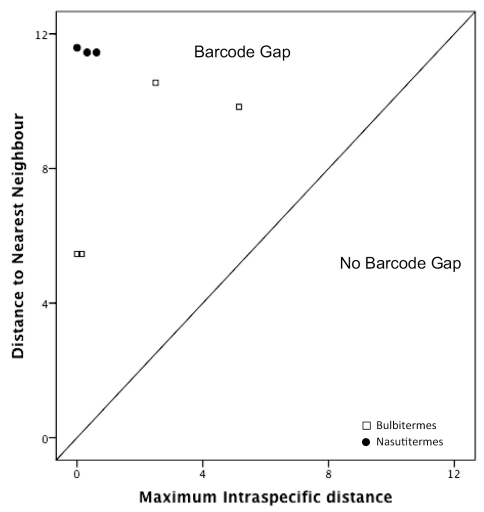
**Figure 26.** Plot of maximum intraspecific divergence against nearest neighbour distance between species (Bulbitermes 4 spp. and Nasutitermes 4 spp.) as identified from morphological characters. All species fall above the 1:1 line, indicating the presence of a barcode gap.

## Discussion

Our taxonomic study has two principle outcomes. First, we identify morphological characters that can be used to systematically identify *Nasutitermes* and *Bulbitermes* from the Sunda region of Southeast Asia. Second, we use these representative taxa to conduct a test case of DNA barcoding in termites. That is, we evaluate how well nucleotide sequence information from the COI barcode region of the mitochondrial genome can resolve species relationships through simple negihbour-joining analysis. We find that the field identification of our eight newly-collected species is possible based on morphological characters alone, and to this effect we present two new functional keys that correspond to each of two genera examined. This morphological classification is consistent with our molecular finding that *Nasutitermes* and *Bulbitermes* have statistically distinct COI profiles. A neighbour-joining tree based on aligned nucleotide sequence shows the complete set of sequenced specimens to cluster by species, and by genus. This clustering is perfect in the sense that no species or genus is mis-classified and no taxon is shown to be paraphyletic with respect to current taxonomy. This combined analysis therefore supports the notion that *Nasutitermes* and *Bulbitermes* are evolutionarily distinct, monophyletic genera – a result that substantiates their taxonomic separation by Emerson.

### Soldier of Bulbitermes

We find that the number of antennal articles is an easy character that can be used in the field to distinguish *Bulbitermes constrictus* from *Bulbitermes neopusillus*; 14 articles for the former and 13 articles for the later. Strongly constricted head-capsule behind antennal sockets also helps to separate these two species. Other species pairs are more difficult to distinguish on morphological criteria. For example, the head capsule is very similar between *Bulbitermes constrictus* and *Bulbitermes subulatus*, especially for specimens collected here from central Sumatra.

The development of our bifurcating key (Key 1) revealed several other criteria for distinguishing species pairs. Eleven antennal articles distinguish *Bulbitermes flavicans* from other three species examined here. *Bulbitermes flavicans* also has a head-capsule that is somewhat round and weakly constricted behind antennal sockets. Finally, there is atypical variation in the antennal segmentation pattern of *Bulbitermes singaporiensis*; most specimens have 12 articles, but minorities have 13 articles. Haviland (1898) made this same observation of *Bulbitermes* from Singapore.

### Soldier of Nasutitermes

We find that the width of the head capsule is an easy character that can be used to separate *Nasutitermes matangensis* from other congeners from the region. Holmgren (1913) already noted the substantial variation in body size for this species (in that case, from Malacca, Malaysia), but subsequent authors (e.g. Ahmad 1965; Thapa 1981; Chhotani 1987; Gathorne-Hardy 2001) nonetheless use size to separate *Nasutitermes matangensis* from its congeners. Other observations from our morphological analysis include: *Nasutitermes neoparvus* separates from the other three species by having exactly eleven antennal articles. In addition, the somewhat rounded head capsule in dorsal view and scattered setae are important characters in recognizing the species. Dimorphic soldiers (the largest and smallest soldiers are markedly different in size) can be used to differentiate *Nasutitermes longinasus* and *Nasutitermes longinasoides*. Finally, Holmgren (1913) and Tho (1992) postulated that the presence of setae on or surrounding the nasus are a defining character for *Nasutitermes longinasus*.

### On identifying Nasutitermes and Bulbitermes

Generally, the soldier caste of *Nasutitermes* is monomorphic; it is rarely dimorphic. The soldier head capsule is, however, highly variable in size and shape, and without constriction behind antennal sockets. The rostrum of the *Nasutitermes* soldier caste is conical to cylindrical, and the antenna is with 11-14 articles. The pronotum is saddle-shaped, and legs relatively short with abdomen elongate. *Bulbitermes* was separated from *Nasutitermes* as a distinct new genus based on constrictions behind antennal sockets by Emerson (in Snyder 1949). However, any single characteristic peculiar to this genus has not been found; Emerson and subsequent authors were not able to show even a single characteristic separating it from *Nasutitermes*. Based on our morphological analysis, we retain the *Bulbitermes* genus. It may be defined by the following morphological conditions: soldier monomorphic; head capsule with a constriction behind the antennal sockets.

We also found many specimens from different morphospecies and habitats with the constrictions behind antennal sockets hardly visible, hence not *Bulbitermes*. While other characters also do not correspond with the *Nasutitermes*. Also not any single morphological character is prominent to erect them to a new genus.

### Molecular taxonomy

Our study shows that DNA barcoding holds promise for helping to solve termite taxonomic problems, with nearest neighbout distances far exceeding maximum intraspecific divergence (Fig. 26), at least for the specimens examined here. Even though our study includes only specimens from a narrow geographic and taxonomic range (two genera within a single subfamily), these observed values suggest that termites are generally amenable to mitochondrial gene barcoding, and that sequence information is potentially useful for delineating species on a larger scale (e.g. Bergamashi et al. 2007). This finding is significant given the rapidly changing higher taxonomy that is currently affecting termite systematic classifications and phylogeny (Eggleton 2011; Engel et al. 2009). Barcode data may help to resolve species relationships within diverse clades (e.g. *Nasutitermes* with more than 250) for which global morphology-based taxonomic keys would be intractable.

## Supplementary Material

XML Treatment for Bulbitermes flavicans

XML Treatment for Bulbitermes neopusillus

XML Treatment for Bulbitermes constrictus

XML Treatment for Bulbitermes singaporiensis

XML Treatment for Nasutitermes matangensis

XML Treatment for Nasutitermes neoparvus

XML Treatment for Nasutitermes longinasus

XML Treatment for Nasutitermes longinasoides
